# The Impact of NOD2 Variants on Fecal Microbiota in Crohn’s Disease and Controls Without Gastrointestinal Disease

**DOI:** 10.1093/ibd/izx061

**Published:** 2018-02-16

**Authors:** Nicholas A Kennedy, Christopher A Lamb, Susan H Berry, Alan W Walker, John Mansfield, Miles Parkes, Rachel Simpkins, Mark Tremelling, Sarah Nutland, UK IBD Genetics Consortium, Julian Parkhill, Chris Probert, Georgina L Hold, Charlie W Lees

**Affiliations:** 1 GI Unit, Institute of Genetics and Molecular Medicine, University of Edinburgh, Edinburgh, UK; 2 IBD Pharmacogenetics Group, University of Exeter, UK; 3 Institute of Cellular Medicine, Newcastle University, UK; 4 Gastrointestinal Research Group, University of Aberdeen, Aberdeen, UK; 5 Pathogen Genomics Group, Wellcome Trust Sanger Institute, Hinxton, Cambridgeshire, UK; 6 Microbiology Group, The Rowett Institute, University of Aberdeen, Aberdeen, UK; 7 Dept of Gastroenterology, Royal Victoria Infirmary, Newcastle, UK; 8 Dept of Gastroenterology, Addenbrookes Hospital, Cambridge, UK; 9 Cambridge BioResource, Cambridge; 10 Department of Gastroenterology, Norfolk and Norwich University Hospital, Norwich, UK; 11 Institute of Translational Medicine, University of Liverpool, UK

**Keywords:** Crohn’s disease, NOD2, microbiota, genotype

## Abstract

**Background/Aims:**

Current models of Crohn’s disease (CD) describe an inappropriate immune response to gut microbiota in genetically susceptible individuals. *NOD2* variants are strongly associated with development of CD, and *NOD2* is part of the innate immune response to bacteria. This study aimed to identify differences in fecal microbiota in CD patients and non-IBD controls stratified by *NOD2* genotype.

**Methods:**

Patients with CD and non-IBD controls of known *NOD2* genotype were identified from patients in previous UK IBD genetics studies and the Cambridge bioresource (genotyped/phenotyped volunteers). Individuals with known CD-associated *NOD2* mutations were matched to those with wild-type genotype. We obtained fecal samples from patients in clinical remission with low fecal calprotectin (<250 µg/g) and controls without gastrointestinal disease. After extracting DNA, the V1-2 region of 16S rRNA genes were polymerase chain reaction (PCR)-amplified and sequenced. Analysis was undertaken using the mothur package. Volatile organic compounds (VOC) were also measured.

**Results:**

Ninety-one individuals were in the primary analysis (37 CD, 30 bioresource controls, and 24 household controls). Comparing CD with nonIBD controls, there were reductions in bacterial diversity, *Ruminococcaceae*, *Rikenellaceae*, and *Christensenellaceae* and an increase in *Enterobacteriaceae*. No significant differences could be identified in microbiota by *NOD2* genotype, but fecal butanoic acid was higher in Crohn’s patients carrying *NOD2* mutations.

**Conclusions:**

In this well-controlled study of *NOD2* genotype and fecal microbiota, we identified no significant genotype-microbiota associations. This suggests that the changes associated with *NOD2* genotype might only be seen at the mucosal level, or that environmental factors and prior inflammation are the predominant determinant of the observed dysbiosis in gut microbiota.

## INTRODUCTION

The precise etiology of Crohn’s disease (CD) remains unknown. However, the key pathogenic process involves an inappropriate immune response that results in bowel inflammation and damage. The targets of this response are thought to be antigens derived from constituents of the microbiota, a view supported by the benefits of altering the microbiota^1^ or physically diverting the fecal stream.^2^ Further, 16S rRNA gene sequencing has shown that the microbiota in inflammatory bowel disease (IBD) is abnormal and characterized by reduced diversity with fewer *Firmicutes* species present.^3^ The direction of causality between IBD and alterations in microbiota remains incompletely understood, as does the question of whether overall dysbiosis or specific taxa are most important. Recent research also has emphasized the functional aspect of the gut microbiota through measurement of microbial metabolites such as the volatile organic compounds (VOC) present in feces.^4^

The last few years has seen rapid advances in the genetics of IBD as a result of large cohort genome-wide association studies (GWAS) of cases and controls. Over 200 IBD susceptibility loci have now been reported.^5–7^ For some loci, the disease gene and associated point mutations are known (eg, *NOD2* and *ATG16L1*). *NOD2* has the largest effect, and a large recent subphenotype-genotype analysis has confirmed that *NOD2* is strongly associated in particular with ileal CD.^8^ Viewed alongside other functionally interrelated genes that have been associated with CD (eg, *ATG16L1*, *IRGM*, and *XBP1*), an impaired capability of the host to regulate microbial constituents consistently emerges as a major common theme.

NOD2 is a cytosolic pattern recognition receptor (PRR) that is a key player in immunity to intracellular bacteria and inflammatory responses. NOD2 recognizes muramyldipeptide (MDP), a ubiquitous component of bacterial cells walls, and its stimulation leads to induction of autophagy in human cells.^9^ Variants of *NOD2* associated with CD are mutated in the ligand recognition domain and fail to induce autophagy on MDP triggering, which results in aberrant bacterial handling and antigen presentation in these cells.^10–12^ NOD2 possesses other antibacterial effects, including the ability to prime human dendritic cells (DCs) to promote T-helper 17 (Th17) responses (via *NOD2*-induced-expression of IL-23 and IL-1)^13^ ,and the ability to induce antimicrobial peptide defensins in the intestine.^14^ If expression of CD-variant *NOD2* leads to dysregulated bacterial destruction within the cells in which it is expressed, bacteria may persist abnormally in the mucosa and activate tissue inflammation in these sites.

However, approximately 11%–14% of white Europeans are heterozygous and 0.4%–0.9% homozygous or compound-heterozygous for CD-risk-variant *NOD2* but remain healthy, which reinforces the role for coexistent genetic or environmental factors in initiation of CD.^15,16^ The association of defective antibacterial mechanisms with CD-associated polymorphisms in *NOD2* suggest that the presence of these variants may influence the nature of the microbiota over time. This in turn might either lead to a critical dysbiotic state being reached, or the presence of specific microbes emerging to initiate the cycle of inflammation observed in disease. For example, altered release of antibacterial peptides from variant-*NOD2*-expressing Paneth cells, defective Th17 responses, or defective autophagic bacterial processing in the gut mucosa could change gut bacterial burden or species diversity.

Little is known of the nature of the microbiota in the presence of *NOD2* mutations. Human studies to date have been limited in scope due to small numbers of individuals homozygous for *NOD2* mutations without accurate matching of controls. Frank and colleagues revisited the dataset from their index 2007 study on the microbiota in IBD, stratifying patients retrospectively for *NOD2* and *ATG16L1* genotype. Due to its retrospective design, this study is severely constrained by limited power; despite this, they observed clear shifts in microbial composition as a result of genotype.^17^ The aim of the current study was to prospectively define the role of *NOD2* genotype in influencing the nature of the host microbiota in health and in CD.

## METHODS

**FIGURE 1.**
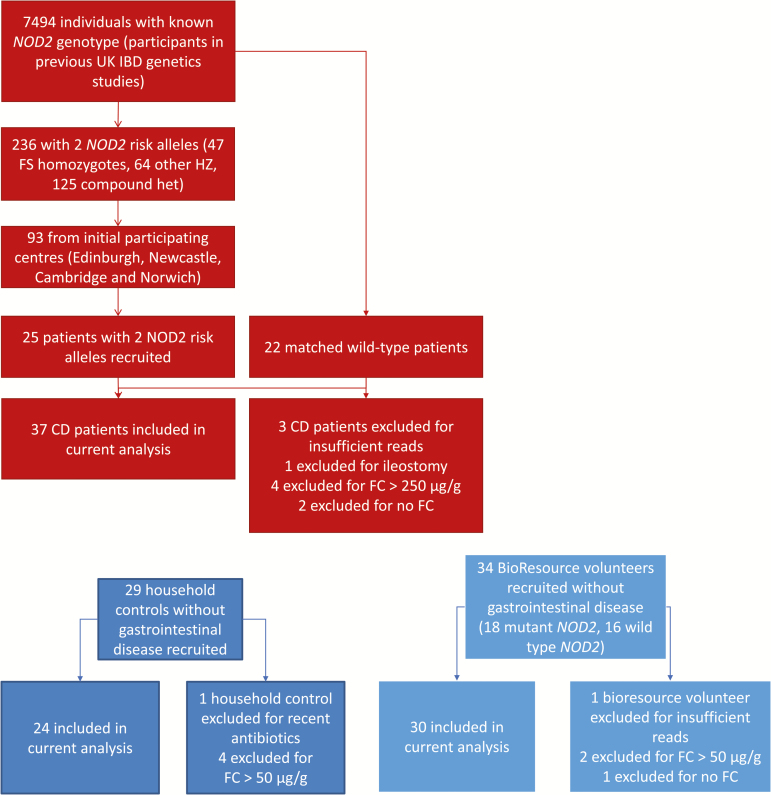
Flow diagram of recruitment.

Individuals with CD of known *NOD2* genotype were identified from the UK IBD genetics consortium (Fig. 1). Patients were selected if they carried 2 copies of the CD-associated *NOD2* variants [ie, homozygotes or compound heterozygotes for R702W (rs2066844), G908R (rs2066845), or L1007fs (rs2066847)]^18,19^ as measured using genotyping arrays for the original genetics studies in which they had been involved (Affymetrix GeneChip 500^20^ and ImmunoChip^6^). Patients were recruited if they were deemed by their treating physician to be in clinical remission. Each *NOD2*-mutant patient was matched to a homozygous *NOD2*-wild-type patient. Exclusion criteria for CD patients included antibiotics within the months before recruitment, active CD (by physician global assessment), and presence of an ileostomy. For all CD patients, a household control was approached (usually an unrelated spouse). Healthy volunteers stratified by the same *NOD2* variants were recruited from the Cambridge BioResource.^21^ The Cambridge BioResource is a panel of around 16,000 volunteers, both with and without health conditions, who have previously submitted DNA for genotyping. Participants can be approached for studies on the basis of genotype and phenotypic characteristics. Volunteers from the BioResource had no known gastrointestinal diagnosis and had not taken antibiotics in the preceeding 3 months. All study participants had fecal calprotectin (FC) measured by a standard ELISA (Calpro AS, Norway). CD patients with FC >250 µg/g and controls (household or BioResource) with FC >50 µg/g were excluded from further analysis.

Clinical data including medical and surgical history, smoking status, medication history, antibiotic use, probiotic use, weight, height, Montreal disease location, and behavior^22^ were collected using a patient questionnaire, interrogation of the medical record, and use of previous phenotype information recorded on the IBD cohorts as part of a rephenotyping exercise.^8^ Probiotic and medication use were documented at the time of sampling. Participants with missing data were excluded from the analysis of that specific datapoint.

Fecal samples were collected from each study participant using the Fisher Fecal Commode Collection Kit. The collection container was held in the toilet bowl using the supplied trivet, and whole fecal samples collected without contamination by urine. Samples were kept cold using phase-change refrigerant gel packs and processed within 24 hours of collection. This short period of storage is not expected to significantly influence molecular estimation of microbial community composition,^23^ nor the VOC profile (unpublished data). Each sample was thoroughly mixed and aliquots were transferred into lysing matrix E tubes (MP Biomedicals, Santa Ana, CA, USA) for subsequent DNA extraction, head space vials (Supelco, Bellefonte, PA, USA) for (VOC) analysis, and universal containers for fecal calprotectin analysis. Samples were stored at −80 °C before shipping to a central processing laboratory in Aberdeen, UK, where DNA was extracted within 1 month of collection.

Household controls had not been previously genotyped. Saliva samples were acquired using Oragene kits (DNA Genotek, Ottawa, Canada). DNA was extracted following the manufacturer’s protocol and was genotyped for the 3 CD-associated *NOD2* variants listed above using TaqMan assays (Applied Biosystems, Carlsbad, CA, USA). Where a genotype was not determined, the allelic discrimination plots were examined manually to ensure homozygotes for the minor allele had not been missed. For the purposes of analysis of the household controls, missing genotypes were inferred to be wild-type genotypes.

Ethical Considerations:

Ethical approval was granted by the North of Scotland Research Ethics Committee (reference 12/NS/0050). All participants provided written consent.

### DNA Extraction

For each fecal sample, an approximately 400 mg aliquot was placed in a lysing matrix E tube and 978 µl of sodium phosphate buffer and 122 µl MT buffer were added to each tube and vortex mixed. This then was processed using the FastDNA SPIN kit for Soil following the manufacturer’s instructions (MP Biomedicals) as described previously.^24^

### PCR Amplification and Sequencing

The V1-V2 region of the 16S rRNA gene was amplified using 27F and 338R primers.^25^ The primers were designed with the Illumina adapter sequences already included and with 1 of 200 barcode sequences included in the 338R reverse primer, thus avoiding the need for a separate step to add the adapter sequences and barcode. Twenty cycles of polymerase chain reaction (PCR) amplification were performed using the Q5 polymerase kit following the manufacturer’s instructions (New England Bio, Ipswich, MA, USA). Postamplification, samples were quantified using a Qubit fluorometer (Thermo Fisher, Waltham, MA, USA) and then pooled to obtain equimolar concentrations.

Sequencing was performed using an Illumina MiSeq sequencer using Illumina V2 chemistry and paired-end 2 × 250 base pair reads. Initial sequence data processing was performed in the Illumina MiSeq Reporter to demultiplex samples and strip adapters and primers and sequence data were exported in the FASTQ format.

### Bioinformatics Analysis

The 16S rRNA gene sequence data were further processed using mothur^26^ following the MiSeq SOP.^27^

Alignment and classification were done against the SILVA v119 reference set.^28^ Community structures were compared using trees generated using Jaccard and Yue Clayton distance metrics after subsampling to 3943 reads per sample. The trees were then plotted graphically using the Interactive Tree of Life.^29,30^ Trees were compared using the parsimony command within mothur. Subsequent statistical analysis was done in R 3.2.2 (R Foundation for Statistical Computing, Vienna, Austria). Microbial diversity was assessed using inverse Simpson and compared using a Mann-Whitney *U* test.^31^ Comparisons at the family, genus, and operational taxonomic unit (OTU) level were done using Mann-Whitney *U* tests for binary comparisons and corrected using Holm’s method.^32^ The 12 most abundant families were selected for plotting graphically.

### Volatile Organic Compound Analysis

VOC data were generated using previously described methodology.^33^ Briefly, gas chromatography-mass spectroscopy (GCMS) was used to quantify the metabolites in the headspace gas taken from vials containing an aliquot of participants’ feces. The raw GCMS data were processed using AMDIS (National Institute of Standards and Technology, Gaithersburg, MD, USA). Compounds detected in fewer than 20% of the study population were filtered out. The resultant ion intensity data were log transformed and the limma package used to facilitate running multiple linear models including disease status and *NOD2* genotype as covariates.^34^*P* values were corrected using Holm’s method.

## RESULTS

Out of the 110 individuals recruited, 91 were used in the primary analysis (Table 1). Reasons for exclusion are shown in Supplementary Table 1. There were 37 CD patients (57% *NOD2* mutant), 30 bioresource volunteers (58% *NOD2* mutant), and 24 household controls. All were of white European ethnicity. There were no differences in phenotype within the CD patients by *NOD2* status (Table 1). Five of 21 genotyped household controls with genotype information had single CD-associated-*NOD2*-associated mutations. Three of these had a first degree relative with CD.

**Table 1:** Study Demographics

A: Whole Cohort					
		CD Patients (n = 37)	Bioresource Controls (n = 30)	Household Controls (n = 24)	*P*
Sex: Female		23 (62%)	16 (53%)	9 (38%)	0.18
Age/years		53 (44–65)	60 (52–64)	51 (43–61)	0.30
BMI		23.5 (21.7–27.2)	25.4 (23.0–27.5)	25.0 (22.8–28.2)	0.12
Probiotic use		3 (8%)	3 (10%)	0 (0%) (1 not recorded)	0.37
Antibiotics within past 12 months (but >3 months)		17 (47%) (1 not recorded)	4 (13%)	9 (39%) (1 not recorded)	0.01
Smoking	Current	5 (14%)	1 (3%)	0 (0%)	0.03
	Ex	18 (49%)	8 (27%)	12 (52%)	
	Never	14 (38%)	21 (70%)	11 (48%)	
B: CD subcohort (from UK IBD genetics consortium)					
			Wild- type *NOD2* (n = 16)	Mutant *NOD2* (n = 21)	*P*
Female Sex			10 (62%)	14 (64%)	1.00
Age/years			56 (46–66)	52 (41–64)	0.36
Smoking		Current	2 (12%)	3 (14%)	0.81
		Ex	9 (56%)	9 (43%)	
		Never	5 (31%)	9 (43%)	
Montreal location		L1	9 (60%)	8 (44%)	0.35
		L2	0 (0%)	3 (17%)	
		L3	7 (40%)	7 (39%)	
		Unknown	1	3	
Montreal behaviour		B1	3 (20%)	4 (22%)	0.73
		B2	9 (60%)	8 (44%)	
		B3	3 (20%)	6 (33%)	
		Unknown	1	3	
History of surgical resection for IBD			15 (94%)	19 (90%)	1.00
Current 5-aminosalicylate or sulphasalazine			3 (19%)	7 (33%)	0.46
Current immunomodulator			8 (50%)	5 (24%)	0.17

Data are presented as medians and interquartile range, or numbers and percentages as appropriate. *P* values are Kruskal Wallis for continuous variables and Fisher’s exact test for categorical variables.

The total number of raw reads was 3,410,868, with a median number of reads per sample of 34,302. After quality control and removal of samples with very low read numbers, the remaining samples had a minimum of 3943 reads and median of 20,338. The sequence data are available from the European Nucleotide Archive under Study Accession Number PRJEB21593.

**FIGURE 2.**
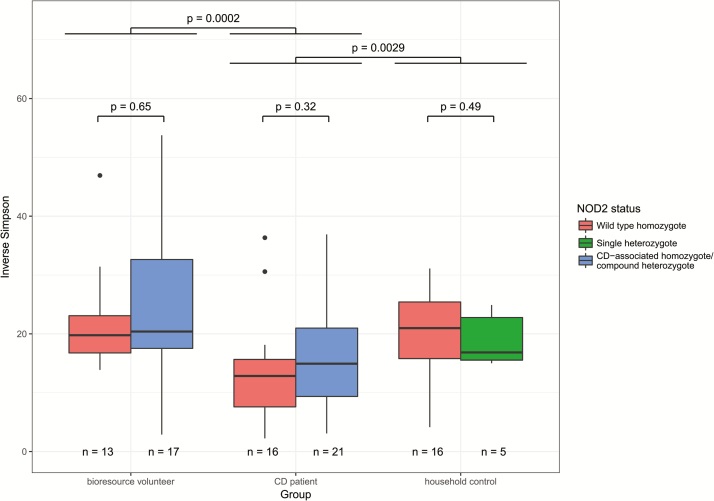
Inverse Simpson index of microbial diversity by *NOD2* status and case type.

**FIGURE 3.**
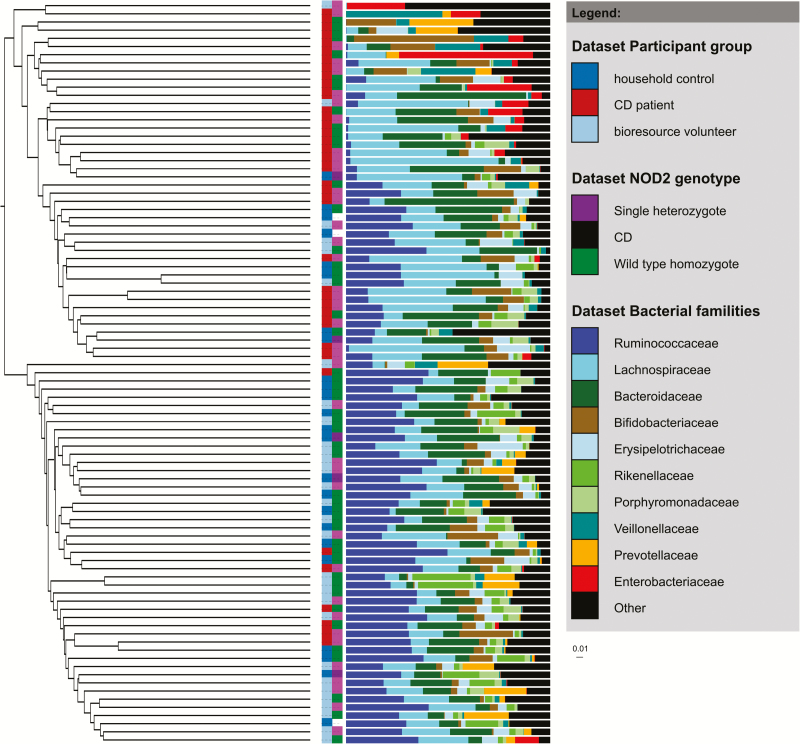
Hierarchical clustering by Jaccard distance metric of the 16S rRNA gene data showing differences by study group and *NOD2* status. The panel on the right shows the relative proportions of the 10 most prevalent bacterial families and the cumulative relative proportion of all other bacteria (shown in black).

There was a significant reduction in diversity (as assessed by calculating the inverse Simpson index) between CD cases and both Bioresource and household controls (*P* < 0.001 and 0.003, respectively, Fig. 2). No difference was observed in diversity by *NOD2* genotype either within the CD cases or the Bioresource controls (*P* = 0.32 and 0.65). Hierarchical clustering using the Jaccard metric demonstrated clustering by CD versus controls in either cohort (*P* < 0.001), but not by *NOD2* genotype (*P* = 0.16 within cases (Fig. 3)). The CD cases also clustered with each other rather than their household controls; indeed the Jaccard distance between cases and their household control was no different from the distance between cases and unmatched household controls (*P* = 0.81, Mann-Whitney *U* test).

**FIGURE 4.**
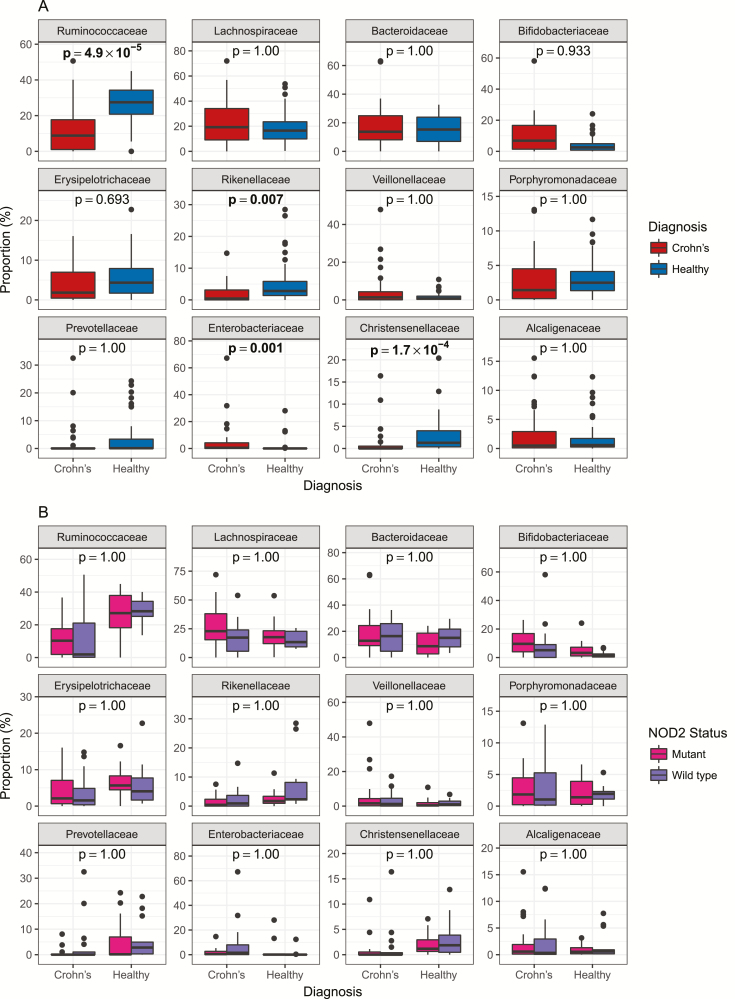
A, Relative abundance of the 12 most prevalent bacterial families in both CD patients and non-IBD controls. *P* values are corrected for multiple testing using Holm’s method across all 59 families seen in the sequencing data. Corrected *P* values < 0.05 are highlighted in bold. B, Relative abundance of the 12 most prevalent bacterial families where samples have been grouped by diagnosis and by *NOD2* genotype. *P* values are corrected for multiple testing using Holm’s method across all 59 families seen in the sequencing data. Mutant *NOD2* is defined here as the presence of 2 CD associated mutations (rs2066844, rs2066845, rs2066847); wild-type *NOD2* is defined as the absence of any of these mutations.

At a family level, there were significant decreases in *Ruminococcaceae*, *Rikenellaceae*, and *Christensenellaceae* (*P* all <0.001 uncorrected and <0.01 corrected), and an increase in *Enterobacteriaceae* (*P* < 0.001 corrected) in samples from CD patients vs controls (Fig. 4A). There were no differences in relative abundance of any bacterial families when stratified by NOD2 status, either within the CD patients or Bioresource controls (Fig. 4B). There also were no differences by genotype at the genus or OTU level in each case, comparisons were made using a Mann-Whitney *U* test with correction for multiple testing using Holm’s method, and no corrected *P* value was less than 0.05.

### Volatile Organic Compound Analysis

For the VOC analysis, there were 314 compounds identified in at least 1 sample, and 198 of those were present in at least 5 CD patients and 5 Bioresource controls.

**FIGURE 5.**
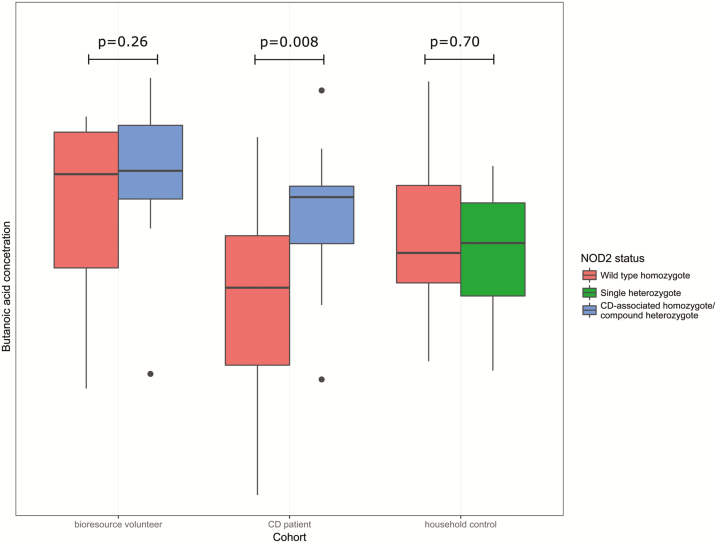
Concentration of butanoic acid stratified by cohort and by *NOD2* status. *P* values shown are uncorrected and are for Mann-Whitney *U* tests by *NOD2* status within each cohort. Mutant *NOD2* is defined here as the presence of 2 CD associated mutations (rs2066844, rs2066845, rs2066847); wild-type *NOD2* is defined as the absence of any of these mutations.

Linear models were constructed for log_2_-transformed data of each compound, with the presence of CD and *NOD2* genotype as the independent variables. These analyses revealed significant reductions in CD patients versus controls in pentanoic acid [log_2_ fold change (logFC) −2.11], piperidinone [logFC −2.43], butanone [logFC −2.19], and acetone [logFC −3.90],Table 2].When looking at the effect of carrying 2 of the previously defined *NOD2* mutations, there was a single significant association after correction for multiple testing using the Holm’s method with butanoic acid (logFC 1.25, corrected *P* = 0.024). On further examination, this VOC was noted to be less abundant specifically in patients with CD with wild-type *NOD2* (Fig. 5).

**Table 2:** Top Volatile Organic Compounds by Presence of CD ^a^

Compound	Log_2_ fold change	*P*	Holm-corrected *P*
Pentanoic acid	-3.29	2.2 × 10^–8^	2.5 × 10^–6^
2-Piperidinone	2.10	1.7 × 10^–7^	2.0 × 10^–5^
2-Butanone	-2.57	3.9 × 10^–7^	4.4 × 10^–5^
Dimethyl sulfide	-2.47	1.3 × 10^–6^	1.5 × 10^–4^
Acetone	-2.25	1.6 × 10^–6^	1.8 × 10^–4^
1H-Indole, 3-methyl-	-4.03	2.3 × 10^–6^	2.5 × 10^–4^
Butanoic acid, 3-methyl-, ethyl ester	2.13	3.5 × 10^–6^	3.8 × 10^–4^
Furan, 2-methyl-	-1.57	1.1 × 10^–5^	0.001
2-Hexanone, 5-methyl-	-1.50	6.1 × 10^–5^	0.006
Butanoic acid, 2-methyl-, ethyl ester	1.90	1.3 × 10^–4^	0.013

^a^Derived from linear model of all CD and non-IBD patients with CD and *NOD2* genotype as covariates.

## DISCUSSION

This prospective study examines the relationship between *NOD2* genotype and the fecal microbiota in human participants stratified by *NOD2* genotype. It further confirms previously identified shifts in gut microbiota in CD patients when compared to non-IBD controls, notably a reduction in obligate anaerobic lineages in tandem with an increase in the facultatively anaerobic *Enterobacteriaceae* family. These changes have previously been described in both inflamed and uninflamed tissue and in both fecal and mucosal samples.^35–38^ However, no significant differences in fecal microbiota were seen when analysed by *NOD2*-status, at any of the taxonomic levels assessed. The present study also includes VOC data and demonstrates the value of having a means to assess the functional aspects of the gut microbiota, and we were able to demonstrate higher butanoic acid concentrations in CD patients with *NOD2* mutations than those without.

Earlier animal studies have shown an association between *NOD2* genotype and gut microbiota. Both Rehman et al and Mondot et al showed reductions in diversity and changes in specific taxa when comparing wild-type and *NOD2* knockout mice.^39,40^ However, more recently, Shanahan et al conducted experiments where the knockout and wild-type mice were cohoused and failed to demonstrate a *NOD2* genotype-specific effect on gut microbiota. They concluded that the cage environment was more important than genotype. Carmody et al went further and looked at the relative impact of genotype and diet on the gut microbiota in mice; they demonstrated dominant effects of diet, regardless of the underlying host genetics.^41^ Nonetheless, Nabhani et al found *NOD2*-related differences in mucosal microbiota even when *NOD2*-knockout and wild-type mouse embryos were mixed and transferred to wild-type surrogates and were subsequently cohoused.^42^

In humans, others have previously reported an effect of *NOD2* on intestinal microbiota. Knights et al reported results from cohorts comprising a total of 474 individuals with IBD, though not stratified by *NOD2* status.^43^ They identified an association between 6 causal *NOD2* variants and increased *Enterobacteriaceae* measured in intestinal biopsies. Of note, they were able to identify similar patterns in ulcerative colitis patients with *NOD2* mutations, suggesting that the observed effect is not just one of disease phenotype. However, in a network of associations between bacterial taxa, host, and environmental factors, the effect of *NOD2* genotype was only modest compared to antibiotic usage, immunosuppressants, biopsy location, and cohort of origin. Li et al reported differences in intestinal biopsy microbial profile related to *NOD2* genotype alongside disease phenotype, with an increase in the *C. coccoides-E. rectales* group in patients with ileal CDcarrying a risk *NOD2* allele.^44^ More recently, Imhann et al reported an interaction between an IBD genetics risk score that included *NOD2* variants and the fecal microbiota, although the impact of *NOD2* on its own was not described.^45^

Strengths of this present study include the use of patients and nonIBD controls of known *NOD2* genotype, with close matching of the phenotypic characteristics across genotypes. Establishing the causal relationship between the gut microbiota and IBD remains challenging; intestinal inflammation is well established as a cause of dysbiosis.^46^ The study excluded participants with either clinical or biomarker evidence of active disease, reducing the possibility of confounding by disease activity. Although the use of patients in remission will have removed one source of variability, it is also possible that the effects of *NOD2* are manifest during active disease. With regards to limitations, this study explores only the changes in gut microbiota in the fecal contents, which are unlikely to fully reflect changes at the mucosal level, particularly in the terminal ileum where one might expect *NOD2* to exert its strongest effect. This reflects the difficulty in accessing colonoscopic biopsy samples in a cohort of non-IBD controls and patients in remission. Although only a single VOC was significantly different by *NOD2* status, this suggests a possibile difference in metabolically active bacteria not well-represented in fecal samples. The patient cohort also had well-established disease, with a history of surgical resection in most participants. This may reflect a higher risk of surgical resection in patients with *NOD2* mutations, noting that the wild-type controls were matched using this phenotype among others. Shotgun metagenomic analysis might have facilitated detection of differences at the species or gene level between cohorts that could be missed with 16S rRNA taxonomic analysis.

## CONCLUSION

This study confirms associations between altered fecal microbiota and Crohn’s disease, but failed to identify any differences in microbiota between individuals stratified by NOD2 genotype. Future studies should explore the relationship between *NOD2* genotype and ileal-associated bacteria, ideally using either cohorts again stratified by genotype or very large cohorts to generate adequate numbers of individuals carrying 2 disease-associated mutations. Large cohort studies also offer the opportunity to perform more extensive genotype-microbiota-phenotype analyses, which should lead to a better understanding of these complex interactions.

## SUPPLEMENTARY DATA

Supplementary data are available at *Inflammatory Bowel Diseases* online.
